# Drone Routing for Drone-Based Delivery Systems: A Review of Trajectory Planning, Charging, and Security

**DOI:** 10.3390/s23031463

**Published:** 2023-01-28

**Authors:** Asif Mahmud Raivi, S. M. Asiful Huda, Muhammad Morshed Alam, Sangman Moh

**Affiliations:** Department of Computer Engineering, Chosun University, 309 Pilmun-daero, Dong-gu, Gwangju 61452, Republic of Korea

**Keywords:** drone-based delivery, drone logistics, drone routing, package delivery, unmanned aerial vehicle, trajectory planning, charging, security

## Abstract

Recently, owing to the high mobility and low cost of drones, drone-based delivery systems have shown considerable potential for ensuring flexible and reliable parcel delivery. Several crucial design issues must be considered to design such systems, including route planning, payload weight consideration, distance measurement, and customer location. In this paper, we present a survey of emerging drone routing algorithms for drone-based delivery systems, emphasizing three major drone routing aspects: trajectory planning, charging, and security. We focus on practical design considerations to ensure efficient, flexible, and reliable parcel delivery. We first discuss the potential issues arising when designing such systems. Next, we present a novel taxonomy based on the above-mentioned three aspects. We extensively review each algorithm for drone routing in terms of key features and operational characteristics. Furthermore, we compare the algorithms in terms of their main idea, advantages, limitations, and performance aspects. Finally, we present open research challenges to motivate further research in this field. In particular, we focus on the major aspects that researchers and engineers need to consider in order to design effective and reliable drone routing algorithms for drone-based delivery systems.

## 1. Introduction

Recently, businesses in the logistics and transportation industry have developed rapidly. The emergence of the e-commerce industry has led to a significant revolution due to internet access to the general public as well as novel delivery methods. The volume of parcels to be delivered is predicted to be as high as 262 billion packages by the end of 2026 [1].

Customers also demand improved service in terms of delivery time and cost. Thus, it is an immense challenge for the service providers to ensure the package is being delivered to the destination as fast and as economically as possible. Furthermore, with the significant increase in the number of delivery packages, the freight costs are increasing due to the locations being widely spread throughout the city. Thus, the service providers are trying to find more effective ways to ensure better delivery service at reduced costs. For example, parcels with common destinations can be grouped to reduce transportation cost. The overall transportation of the delivery does not face any difficulties until the packages arrive near the destination. However, the unit cost per transportation increases because every package has its own unique delivery process, and the delivery costs are no longer shared. This is referred to as the “last mile challenge”, which is encountered by many delivery service providers [2,3,4].

Therefore, e-commerce businesses have started exploring innovative delivery strategies to stay competitive and lower their delivery costs for customer retention and growth. An effective solution to the above-mentioned problem can be addressed by adopting an innovative autonomous drone-based delivery technique, which can mitigate the extra cost incurred by the increased delivery constraints. Such autonomous systems can provide reliable, swift, and efficient delivery services, which helps limit the last-mile transportation cost. This is particularly important in remote areas where the package delivery rate is minimal, such as small countryside cities and villages. In addition, the dispersion of delivery destinations adds to this problem. Thus, advanced techniques that consider all the practical constraints are required.

The prospect of drone-based delivery has started to become a possibility for the future e-commerce, logistics industry, and integral part of smart city [5]. Reputed and well-known organizations such as Google, Federal Aviation Administration, and Amazon have started testing the use of drones to deliver parcels and packages to their destinations [6]. Recently, Amazon proposed using a giant flying warehouse that can carry drones, product inventory, and staff to address the limited payload capacity and service range problem [7]. The general drone-based delivery scenario can be envisioned as follows: Once the order of any package/meal has been confirmed, the delivery system assigns a drone to pick the package up from the source and deliver it to the actual destination. Afterward, a drone is selected to perform the mission based on the available battery and destination. When the trip ends, the drone may have several decisions to make, such as whether to recharge the battery, deliver the next parcel, or wait to receive the next parcel [4].

**Figure 1:**
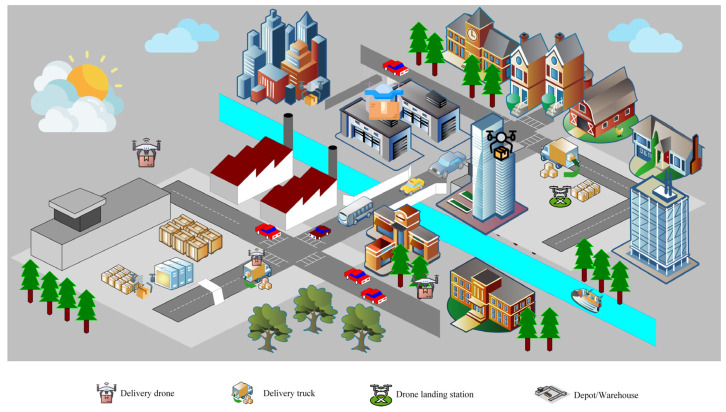
General scenario of a drone-based delivery system.

A general drone-based delivery scenario is presented in Figure 1. As the application of drones is growing popular due to technological advancements, the logistics industry is also trying to yield from the success being brought about by the drones. Thus, the rise of drone-based delivery systems come into picture. Employing drones for the delivery mission provide advantages over traditional logistics in various aspects. In comparison to the conventional truck delivery scheme, the advantages of drone-based delivery are as follows [6]: (1) Faster delivery can be ensured by reducing the delivery time and travel distance. (2) Cost can be reduced because drones are cheaper than trucks. (3) Delivery can be ensured even in emergency situations such as floods and earthquakes [8]. (4) This system can provide ‘last-mile’ delivery or delivery at the doorstep of the customer. (5) It reduces carbon emissions as drones do not heavily rely on fossil fuels.

Although the overall prospect may sound promising, the underlying scenario of drone-based delivery requires further investigation. Numerous constraints have a direct impact on the performance of drone-based delivery systems. First, drones have limited battery capacity. Thus, they can only fly for a limited time which may be insufficient for the drone to cover a certain area. Thus, charging stations (CSs) must be established to guarantee longer flying time. The CSs can be utilized as platforms where drones land and have their batteries changed or recharged. Second, currently, drones cannot carry bulky packages or travel longer distances due to limited battery capacity. Third, due to extreme weather conditions (e.g., heavy wind, snow, and rain) drone flights can significantly impact the performance of drone-based delivery systems. Finally, the service providers need to ensure minimum delivery cost because most users would be unwilling to use the service if they perceive the delivery cost to be unaffordable. Thus, the service providers need to consider various costs in terms of drone maintenance, recharging, and operational strategies. Thus, in the logistics industry, every company willing to integrate drone-based delivery service is faced with numerous challenges and important issues in deploying the system at a reduced cost. These include delivery mission design and planning, drone routing and charging, and multiple parcel deliveries on a single mission. These dynamic factors make the overall planning more complex and challenging.

We identified that the technical issues for drone routing in drone-based delivery systems are coupled with trajectory design, charging strategy, and security aspect. The trajectory design considers efficient planning of the waypoint and visiting order of customers or delivery locations according to drone’s flight time. Since drones have the limited flight time due to the constrained battery capacity for long distance and, sometimes, they carry out multiple deliveries in a single mission, a charging strategy is required. Thus, the charging strategy considers the battery replacement or replenishment strategy to extend the drone flight time by placing charging stations at optimal places. Finally, the security aspect ensures the required security and privacy in drone-based delivery systems as drones contain the sensitive information of both proprietors and customers (i.e., user/product information and trajectory data).

In drone-based delivery systems, trajectory planning can be classified based on the delivery method type. Researchers have considered single drone-based delivery [9], truck-drone collaborative delivery [10], public transport and drone collaborative delivery [11], and multi-drone-based delivery with nearby CSs for multiple customers [12]. The overall trajectory or route planning to visit the customers/delivery points or CSs are derived as different variants of the travelling salesman problem (TSP) [10,12,13,14]. Many mathematical models such as mixed integer linear programming (MILP) [12], heuristic method [15,16], and reinforcement learning [6] have been adopted to obtain a sub-optimal solution under different objectives considering several constrains. The main objective of trajectory planning in drone-based delivery systems is minimizing the average delivery time [11] and maximizing coverage area to serve maximum number of customers in a single charge [17]. The major constraints in trajectory planning are limited battery of drones and their constrained payload capacity, limited number of drones and CSs for the given area, restriction on the placement locations of CSs, presence of static and dynamic obstacles, restricted area for flying, threshold time to complete the delivery, and different uncertainties based on the weather conditions.

In order to address the above-mentioned challenges, an appropriate charging strategy for the drone-based delivery systems is necessary. Therefore, some studies formulated joint charging and routing strategies to meet this demand [12]. It is crucial to obtain the optimal number of CSs with suitable locations according to the distribution of delivery points to extend the flight time of drones. Diverse strategies have been attempted to meet this demand. Generally, for long-distance delivery missions, CSs enabled with wireless power transfer are considered. In contrast, in truck-drone collaborative delivery, drones are considered last-mile delivery vehicles, and battery replacement is considered a convenient solution to reduce charging time and minimize delivery time. Thus, in a typical truck-drone-based delivery systems, trucks provide the charging facility. Some approaches consider the use of public transport to provide charging to the drones [18] and as a carrier to bring the drones to a location that is closer to the customers in remote places [11].

To keep the customer integrity in terms of delivery data and trajectory data, appropriate security measures must be implemented. Moreover, due to the recent booming of the e-commerce platform, drone-based delivery systems are being promoted as an urgency to serve as a faster delivery system. Thus, hacking of a drone containing sensitive information related to both proprietor and customer puts a huge risk for both parties involved [19]. Additionally, the leakage of monetary information, transaction record, and other information of both parties would jeopardize the whole drone-based system. Many blockchain based techniques, where the information is stored in blocks, and encryption-decryption approaches are being implemented to solve this issue [20]. The different aspects of the routing problem are detailed in the upcoming sections.

Thus, in this review, we aim to identify the main design problems that arise when designing drone routing techniques in drone-based delivery systems. We achieve this by comprehensively reviewing the existing drone routing algorithms. We categorize the existing drone routing techniques in terms of three aspects: trajectory planning, charging, and security. We then detail their key features and operational characteristics. Subsequently, we compare the drone routing techniques in terms of main idea, advantages, limitations, performance aspects, and outstanding features. Moreover, we provide future research and development challenges that may have crucial impact on the future drone-based logistics systems.

### 1.1. Related Surveys

In recent years, numerous efforts have been made to address the existing drone-based logistics systems, as summarized in Table 1. Most existing reviews have emphasized the different aspects of drone-based delivery systems to provide a holistic review. Next, we discuss and summarize the reviews that cover the current research progress on drone routing problem for designing drone-based logistics systems.

In [4], the authors investigated the major issues faced while designing and modeling the drone-based delivery systems and their potential solutions. In addition, they provided the current scenario of parcel delivery using drones with respect to the existing literature. However, this study presents a general overview of the existing literature and does not discuss the major design issues in sufficient detail. In [21], the authors discussed the existing techniques for drone-based delivery from an energy management perspective. The authors discussed several ways to provide energy to the drone during the delivery mission such as battery swapping, utilization of laser beam based charging, and tethered UAVs. In [22], the authors emphasized the operational planning aspect of the drone-based delivery systems. They presented a comprehensive review with a unique classification of the existing logistics systems. This classification was made in terms of various operational factors such as supporting vehicles, number of depots, and touchpoints. Although their presented classification is unique, their study focuses only on the operational aspects and ignores other major aspects of drone-based delivery such as energy management techniques, routing techniques, and security issues in drone-based delivery systems.

In contrast with the abovementioned works, in [23], the authors presented a unique perspective by emphasizing the public mindset towards adopting the drone-based delivery service. The authors addressed the user mindset on drone-based logistics and their intention on adopting such services. They concluded that people’s mindset varies with their residing area. In [24], the authors focused on hybrid delivery techniques that included trucks and drones. The authors provided background knowledge of the recent progress on the drone-based delivery systems, followed by a novel classification of truck-and-drone-based delivery systems. The comparison of different studies is also given in terms of design objectives, solution techniques, role of the vehicles, and overall system configuration. In particular, the authors discuss three different configurations of hybrid truck- and drone-based delivery systems: single truck—single drone, single truck—multiple drones, and multiple truck—multiple drones. In [25], the authors surveyed drone scheduling techniques for various purposes such as parcel delivery, recharging considerations, and monitoring, which are equally important for designing drone-based logistics systems. They summarized the existing studies in terms of various crucial parameters such as objectives, number of drones and vehicles, solution of the problems, and innovative ideas. They focused only on the drone scheduling aspect and ignored other dominant concerns in drone-based logistics.

In [26], the authors investigated why customers are reluctant to adopt drone-based food delivery systems. Specifically, the authors designed a unique framework to explain several barriers such as value, risk, experience, and tradition in customers’ unwillingness to adopt drone-based food delivery. This survey is particularly important because it reveals the perception of customers. This can significantly improve the drone-based food delivery service. To understand the customers’ mentality towards adopting drone-based logistics systems in a pandemic situation such as COVID-19 [27], the authors in [28] particularly focused on the determining factors that encourage or discourage the adoption of such services. In particular, the authors aimed to investigate the customers’ mindset to enable the policymakers to decide whether they should shift towards drone-based delivery from the traditional delivery system. The main limitation of this study is that it was conducted on data collected from a very small region, which limits its generalization capability.

**Table 1:** Comparison of existing surveys.

Paper	Year	Design Issues	Trajectory	Charging	Security	Challenges	Major Focus
[4]	2022	✗	✗	✗	✗	√	Current state of delivery. Current research of drone-based delivery.
[21]	2022	✗	✗	√	✗	✗	Energy management strategies
[22]	2021	√	√	✗	✗	√	Operational design characteristics. Future research direction.
[23]	2018	✗	✗	✗	✗	✗	Factors affecting the adoption of drone-based delivery service. Factors determining the adoption of drone-based delivery service by the public
[24]	2022	√	√	✗	✗	√	Review of the hybrid truck-drone-based delivery. Classification based on configuration and objective.
[25]	2022	✗	✗	✗	✗	✗	Fundamental overview of drone scheduling. Drone scheduling for drone-based delivery.
[26]	2022	✗	✗	✗	✗	✗	Fundamental overview of the drone-based food delivery. Barriers that prevent the adoption of drone-based delivery.
[28]	2022	✗	✗	✗	✗	√	Overview of drone-based food delivery. People’s tendency to adopt food delivery during the pandemic.
[29]	2020	✗	√	✗	✗	√	Technological background. Drone routing techniques. Conclusion and future challenges.
Our work	2023	√	√	√	√	√	Design issues of drone routing in drone-based delivery systems. Review of existing drone-routing algorithms for drone-based delivery, focusing on three major aspects of trajectory planning, charging, and security Comparison of the state-of-art drone routing algorithms for drone-based delivery. Potential future research and development challenges.

Based on the above discussion, it can be observed that the existing surveys cover different aspects of drone-based delivery systems. However, the major design issues are mostly overlooked in the existing studies, which include drone trajectory, delivery time, energy management, depot placement, distribution of delivery points, payload constraints, and priority-based deliveries. Thus, there is a need for a survey that covers the major design issues while pointing out the recent developments on the overall aspects of the drone-based delivery systems. Thus, in this study, we first provide an overview on the design issues of the existing drone-based delivery systems. We then classify the existing drone-based delivery techniques and review them in terms of their major characteristics. Next, we compare the existing drone-based delivery techniques in terms of their advantages, limitations, main idea, innovative features, performance aspects, and constraints. Finally, we provide open research issues to motivate further research in this field. The major contributions of this study are summarized in the next subsection.

### 1.2. Contributions of This Study

This study aims to provide a comprehensive survey of the existing drone routing algorithms for drone-based delivery by covering the dominant aspects, design issues, and research challenges. The major contributions of this study are summarized as follows:

- First, we discuss several crucial design issues of drone routing in drone-based delivery systems. This discussion includes the important issues for implementing such systems.
- Next, we provide a novel taxonomy where we emphasize three major aspects: trajectory planning, charging, and security algorithms for designing drone routing algorithms in drone-based delivery systems.
- To provide the readers with an overview of the current progress of drone routing in drone-based delivery systems, the existing drone routing algorithms are extensively reviewed in terms of their design model, optimization objective, assumptions, and operational strategy.
- Further, we compare the drone routing schemes in terms of their main idea, optimization objective, constraints, advantages, limitations, and performance.
- Finally, important research and development challenges are discussed to motivate further research in this domain.

### 1.3. Organization of the Paper

**Figure 2:**
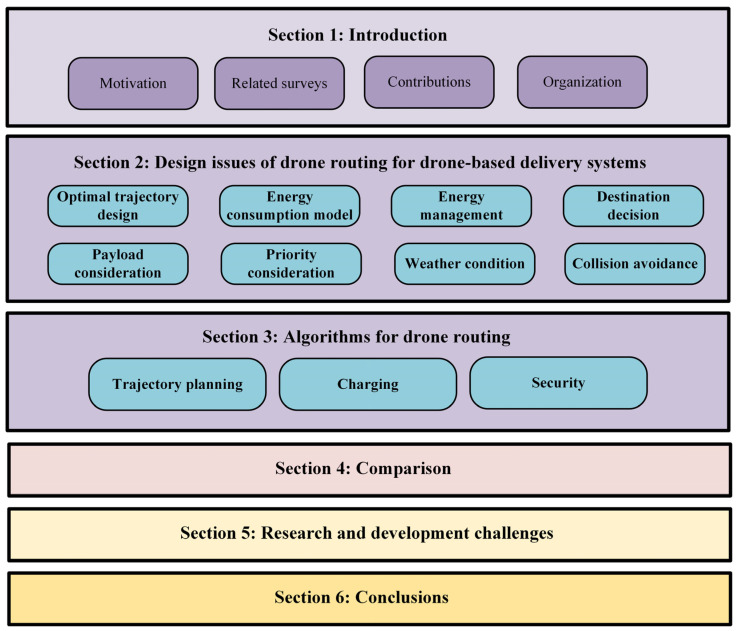
Outline of the survey.

This survey consists of seven sections, as shown in Figure 2. Section 2 discusses the major design issues of drone routing for drone-based delivery systems. In Section 3, the existing drone routing algorithms are classified into trajectory planning, charging, and security algorithms and, next, each of them is extensively reviewed to provide the readers with a clear view of the operational principles and characteristics of drone-based delivery systems. In Section 4, we compare the drone routing algorithms in terms of their advantages, limitations, main idea, and innovative features. In Section 5, we discuss open issues and challenges for future research and development. Finally, the conclusions of this review are drawn in Section 6.

## 2. Design Issues of Drone Routing for Drone-Based Delivery Systems

In this section, we present the major design issues that must be considered while designing a drone-based logistics system. There has been significant research trying to solve the crucial issues to enhance the performance of drone-based delivery systems. However, with additional demand and dynamic environment conditions, the design issues are growing increasingly complex, due to which the service providers are struggling to design economical and effective delivery systems [30].

### 2.1. Designing Optimal Trajectory to Reduce Delivery Time

Trajectory design is one of the major challenges in drone-based delivery systems as it helps reducing the total travel time. Due to diverse delivery locations and charging points, it is often challenging to find the optimal trajectory that can minimize the overall delivery time and enable the drone to successfully fly back to the depot [31]. Analyzing the drone’s trajectory data and optimizing it is an NP-hard problem, where NP stands for non-deterministic polynomial-time. As the number of depots and customer points increases, the problem of searching for optimal trajectory becomes extremely complex [6]. Thus, significant efforts should be made to avoid such shortcomings. 

### 2.2. Drone Types and Energy Consumption Model

Energy consumption is one of the most crucial metrics for drones due to their limited payload capacity, constrained battery, and limited flight time [32]. To precisely estimate the drone flight time and state of battery, a sufficiently accurate mathematical model for drone energy consumption is required [33]. It must consider the mechanical structure of the drone (such as the number of rotors) according to its type, effects of wind, gravity, attainable velocity, traveled trajectory, altitude, and current payload [12,13]. Thus, due to the limited battery lifetime and surrounding environment conditions, the energy of the drone can drain drastically during the delivery of a parcel, which would result in mission failure and cause serious damage to the reputation of the logistic service provider. Thus, it is indispensable to consider determining factors such as how to deliver goods to the final destination in case the energy of drone is affected during its mission [34].

### 2.3. Drone Energy Management

Due to the dynamic environment conditions and increasing delivery constraints, the energy in drone batteries would be insufficient to ensure successful parcel delivery. Thus, it is critical to decide additional ways from which energy can be provided to the drone during the mission. There are several ways in which drone battery can be recharged, such as battery swapping, laser-beam recharging, and charging via wireless power charging pad [21].

### 2.4. Decision on Customer and Delivery Point

In a drone-based delivery mission, the drone can either go to single or multiple destinations. For example, the drone needs to pick up the parcel from a certain location or depot and, after delivering the parcel, it needs to decide whether to wait for the next parcel or fly back to the depot. These considerations depend on the current energy of the drone and the delivery route. Thus, it is crucial to design optimal routing decision-making algorithms that would consider these conditions. Hybrid truck-drone techniques have been proven successful in cases where joint considerations of drone scheduling and routing decision-making have been made considering customers widely distributed over a certain region [10,35].

### 2.5. Payload Consideration of Drones

A higher payload weight is detrimental to the drone-based delivery performance. Since one of the major portions of the drone weight is consumed by the battery, to which the parcel weight is added. With increasing weight, the flight time reduces significantly. Thus, payload constraints should be considered to design an effective and reliable drone-based delivery systems. Studies that consider both payload and battery weight have shown significantly minimized overall cost and flight time for drone-based delivery [13].

### 2.6. Priority-Aware Trajectory Design

In a drone swarm delivering packages, it is important to consider the payload and battery constraints of the individual drones to minimize the overall delivery time. For example, drones with comparatively higher payloads will drain more battery than those with lower payloads. Therefore, some of the drones may consume their energy faster than usual, which would significantly affect the overall swarm performance. Thus, an ideal solution is to prioritize the drones carrying heavier payloads by designing an optimal trajectory to place them in a suitable position and to reduce the number of waypoints they visit [36]. This solution would ensure that these energy-constrained drones consume the least amount of energy by traversing through the optimal waypoints to reduce the overall delivery time, and the package delivery is guaranteed successfully without drone failure. In-flight drone-to-drone charging can also be a potential solution to recharge the drones with limited battery [36].

### 2.7. Weather Conditions

Weather consideration is crucial to ensure drone-based delivery during an emergency. Bad weather conditions, such as high wind speed and rainfall, can significantly affect drone flight. Therefore, the weather data are considered major pre-requisites to ensure reliable drone performance. For example, when the wind speed is significantly higher or rainfall increases, it is better to discontinue the flight, otherwise, the drone would incur significant damage, which would increase the overall cost. Thus, depending on the periodical weather data, such as wind and rain, the system can decide whether it should continue the mission or recall the drone to the depot [37].

### 2.8. Collision Avoidance

Although single drone-based delivery systems are being introduced recently, use of a drone swarm is necessary to meet the increasing delivery requirements. Thus, effective collision avoidance techniques should be considered so that drones do not collide with each other during their flight. In addition, in urban areas, there are numerous high-rise buildings and other obstacles, which makes the overall environment extremely dynamic. In addition, the delivery service needs to be highly efficient and responsive in those areas [38]. To design an effective collision avoidance algorithm, several considerations must be made. These include data transmission latency, computation latency, and control response latency. Even after designing a collision avoidance technique, a severe collision can occur due to the incurred latency. Therefore, these individual latency values should be considered jointly to ensure optimal flight performance [37].

## 3. Drone Routing Algorithms

In this section, we discuss algorithms for drone routing in drone-based delivery systems in terms of three different aspects, namely, trajectory planning, charging strategy, and security, which are crucial for designing effective and reliable drone-based delivery systems. Furthermore, we investigate and discuss each algorithm in terms of different aspects, including its main objective, operational characteristics, advantages, limitations, and performance.

**Figure 3:**
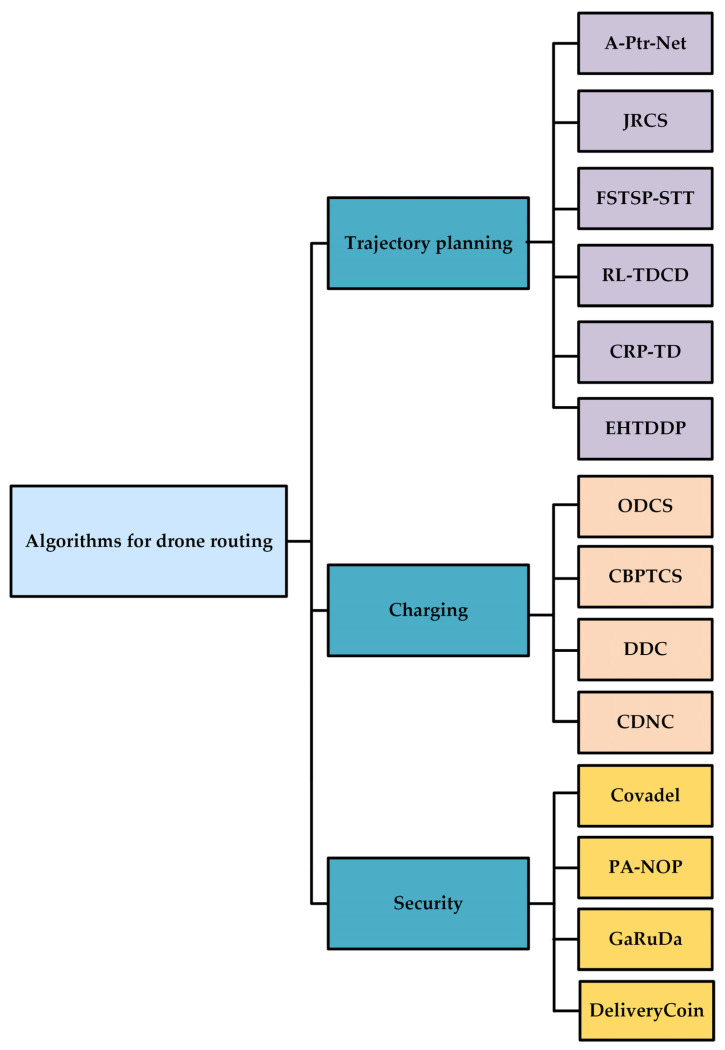
Taxonomy of algorithms for drone routing in drone-based delivery systems: A-Ptr-Net [6], JRCS [12], FSTSP-STT [41], RL-TDCD [42], CRP-TD [15], EHTDDP [16], ODCS [44], CBPTCS [11], DDC [17], CDNC [51], Covadel [56], PA-NOP [57], GaRuDa [58], DeliveryCoin [60].

First, we classify the existing algorithms for drone routing in drone-based delivery systems. That is, we present a novel taxonomy emphasizing three major drone routing aspects for drone-based delivery systems: trajectory planning, charging, and security. The taxonomy is depicted in Figure 3.

### 3.1. Trajectory Planning

One of the major concerns of the drone-based delivery systems is to design the optimal trajectory of drones with maximum possible energy efficiency because drones have limited flight time owing to their limited battery capacity. The term ‘trajectory’ is often interpreted as routing path, waypoint design, and path planning across various literatures. The trajectory of the drone-based delivery system is often formulated as a TSP, where the drone visits each of the customers or waypoints based on the requirement imposed by the proprietor. For drone-based delivery problems, several variants of TSP are formulated to meet the customers’ demand as well as the proprietors’ profit margin. These variants are dependent on payload, battery capacity, and traveled trajectory. In most cases, the trajectory planning of the drone can be classified under three categories: independent single-customer-based drone-only delivery, truck-drone collaborative delivery, and multi-drone-based delivery with nearby CSs.

In independent single customer-based delivery, drones deliver the parcels to the designated customers, one at a time. After the completion of delivery, the drone either comes back to the depot to make a new delivery or goes to a CS for charging. This method is relatively inefficient as drones must charge frequently or return to the depot after making a delivery with available energy to spare. Thus, this approach is drawing relatively less attention from researchers as well as industries. However, new research methodologies and modifications continue to open new pathways toward this short-haul delivery system. 

In the case of truck-drone collaboration, trucks carry high payloads to long-haul destinations. Next, to make ‘last-mile’ delivery where the truck is unable to go any further, a drone is deployed from the back of the truck to deliver the parcel to the customer. These problems are classified as flying sidekick traveling salesman problem (FSTSP) [39], vehicle routing problem with drones (VRPD) [40], and last-mile delivery (LMD) problem [37]. In some cases, the truck or the medium of transport itself acts as a CS to provide the drone with battery backup through a charging facility [18].

For multi-drone-based delivery with nearby CSs, drones leave the depot with the parcel and deliver it to customers, where the customers are grouped or clustered based on the nearby CS. The trajectory is designed so that the drone can make the deliveries and returns to nearby CS for recharging before its battery is completely discharged. This method is discussed further in the drone charging methodologies subsection.

#### 3.1.1. Attention-Based Pointer Network (A-Ptr-Net)

Finding optimal trajectory for drone-based delivery systems is indispensable from a design perspective, and considerable design effort is required from a research perspective. The A-Ptr-Net [6] recognizes the drawbacks of the traditional delivery logistics drone using heuristic approaches when drones need to make multiple deliveries to the customer from depot and return for the purpose of recharging and reacquiring parcel. From the drone trajectory data, the scheme puts additional emphasis or attention on the newest drone trajectory regardless of the discovered distance or distance matrix. The developed model is self-taught and utilizes reinforcement learning to update the distance matrix for the newly updated flight trajectory. In the decoder part of the devised model, a multi-head attention mechanism with a fully connected feed-forward layer is added to further enhance the trajectory data. The novelty of this step is to obtain multiple outputs instead of a single entity to increase the generated data and give attention based on the desired requirement. Thus, a convex function is designed to mitigate delivery error and ensure that the targeted delivery is within a reasonable time frame.

#### 3.1.2. Joint Routing and Charging Strategy (JRCS)

Planning a drone trajectory path for a long delivery mission effectively is a tedious question, considering the limited flight capacity of drones. In [12], the authors recognize this problem and address it by effectively dividing the delivery region into clusters. The centroid of the cluster is chosen as the CS to ensure maximum flight range. The ground users are clustered, and the proposed system ensures that all the customers are visited at least once. The formulated scheme ensures maximum flight distance in a single charge before recharging again. A MILP formulation is used to derive the drone-based delivery routing problem (DDRP), which minimizes the mission time of the drone-based delivery.

#### 3.1.3. Flying Sidekick Traveling Salesman Problem with Stochastic Travel Time (FSTSP-STT)

The concept of truck-drone pair has been on the rising tide to provide logistics support for the last mile. In [41], authors proposed the (FSTSP-STT) scheme to address the parcel delivery problem as a modified FSTSP, which is similar to the vehicular routing problem (VRP), and formulated it using Markov decision process (MDP) by considering the stochasticity of the travel time. The proposed formulated scheme is answered by using deep Q-networks (DQN), which combines reinforcement learning (RL) and deep neural networks, and further refined by the attention mechanism. Moreover, the actor-critic (A2C) network was used to address the problem of dimensionality, which is generally observed in machine learning techniques. This scheme tries to address the fact that the DRL based algorithm performs better than traditional heuristic approaches.

#### 3.1.4. Reinforcement Learning-Based Truck-and-Drone Coordinated Delivery (RL-TDCD)

The scheme proposed in (RL-TDCD) [42] considers an unmanned delivery system where a truck and a drone perform the delivery work in a coordinated manner. The truck acts as CS as well as a landing station for the drone. The truck and drone routing problem was formulated using RL. The formulated delivery problem is initially presented as a mixed integer programing (MIP) problem, which is further broken down into three subproblems to further reduce its complexity. Using k-means clustering, the service or coverage area for the parcel delivery is determined. Moreover, an encoder-decoder framework is designed in addition to RL to address the routing problem. However, the encoder-decoder framework combined with reinforcement learning for solving the routing problem may have difficulties in adapting to more complex scenarios.

#### 3.1.5. Collaborative Routing Problem-Truck and Drone (CRP-TD)

The work in [15] investigates a collaborative truck-drone routing problem for contactless parcel delivery (CRP-TD) during the COVID-19 pandemic. It develops an improved variable neighborhood descent (IVND), which combines the metropolis acceptance criterion of simulated annealing and the tabu list of the tabu search algorithm to solve the formulated problem, which minimizes the delivery time and considers energy consumption of drones. It also integrates the K-means clustering and the nearest neighbor strategy to generate an initial solution. The performance of IVND was evaluated by comparing it with other algorithms on instances with different scales as well as testing several critical factors to verify its robustness. The experimental results demonstrate that IVND has superior performance compared to other benchmarks in terms of both speed and accuracy.

#### 3.1.6. Exact and Heuristic Approaches to Truck-Drone Delivery Problem (EHTDDP)

The proposed algorithm in [16] focuses on coordinated truck-drone delivery problem, in which a traditional delivery truck is combined with a drone to reduce delivery times and costs. A MIP formulation and heuristic approach were proposed to address the FSTSP which is basically TSP with drones. In brief, the MIP formulation addresses the short instances whereas heuristic approach successfully tackles large instances. The MIP formulation yielded better linear relaxation bounds than previously proposed formulations for all instances, while the hybrid heuristic was able to improve over 80% of best-known solutions for large-size instances.

### 3.2. Charging

Owing to the limited battery capacity and extra payload of delivery products, the flight time of the drone is short. The optimal clustering of targeted customers, the drone route planning according to customer order or priority of releasing payload, and the trajectory optimizations can minimize the drones’ flight time and energy consumption. However, to efficiently cover a large area with many customers, drones require a battery charging strategy. Additionally, it is challenging to provide multiple deliveries simultaneously over long distances to support the maximum number of customers within a constrained timeframe by avoiding uncertainties of drone failure due to sudden energy depletion. Since drone-based delivery is mostly considered for delivering goods to remote areas, the drone flight time should be sufficiently large. Additionally, if a single drone can support multiple nearby customers in a single mission with extended flight time, it can deliver the desired product within the given time, which may help achieve customer satisfaction and successfully execute the delivery mission. Therefore, a suitable charging strategy is required to extend the flight time of drones. The charging strategy mainly considers the placement of optimal number of CSs to maximize coverage and drone flight time, and minimize the delivery time and system deployment cost [43].

In order to achieve the above objective, several research ideas have been proposed to charge drone battery while it is executing the delivery mission. Most designs consider the optimal number of CSs and their placement according to the location of customers and hot spot areas [44]. Based on the flight time constraint, drones can recharge their battery, or their battery can be replaced automatically or manually at the CSs [21].

The locations of CSs can be fixed [45] or mobile [46]. Moreover, they may have multiple charging pads to serve multiple drones simultaneously via wireless power transfer [33,47]. Some designs consider collaboration between public transport, dedicated truck-based drone-enabled delivery, and CSs to execute drone-based delivery mission in remote areas [11,48]. To plan a charging strategy, the drone flight time must be calculated precisely by adopting a suitable energy consumption model [12]. Notably, the energy consumption of a drone depends on its type, mechanical structure, weather conditions, payload, drone dynamics, and traveled trajectory. An insightful discussion about drone energy consumption model is given in [34,49,50]. Some innovative research ideas regarding drone charging in a drone-based delivery mission are discussed below, considering their designed model, optimization objective, mathematical model, and constraints.

#### 3.2.1. Optimized Deployment of Charging Station (ODCS)

In [44], the author proposed ODCS to address the issue of finding the optimal number and location of CSs in a given area to serve maximum customers. The placement of CSs prioritizes the most demandable locations, which can be determined based on the delivery frequency or historical data. ODCS considers several constraints in the optimization process, such as limited parcel weight, distance between two CSs according to the flight time of a drone, connection of the CSs till depot according to the drone’s flight range, and threshold coverage ratio of the CSs. In ODCS, initially, it deploys the CSs in a triangular pattern to cover the entire demand area. Next, it recursively removes the extra CSs based on the least demand locations and re-adjusts the locations of other CSs to achieve higher coverage ratio. This re-adjustment process continues until no further removal of CSs is possible, thus satisfying the abovementioned constraints.

#### 3.2.2. Collaboration between Public Transport and Charging Station (CBPTCS)

In [11], the authors proposed CBPTCS considering collaboration between public transport and CSs to execute drone-based delivery in rural areas. From the depot, a drone takes on suitable public transport to reach the best possible closest location of the targeted customers located in distant areas. Next, drones leave the public transport to reach the customers and based on the flight time constraint, drones can utilize CSs to swap their batteries. The main objective of CBPTCS is to minimize the average service delivery time to achieve customer satisfaction, which is carried out by optimally placing CSs. Based on the precise computation of drone flight distance, CSs are initially deployed to construct a fully connected topology considering the connectivity between CSs and all customers. Next, a minimum spanning tree (MST) is constructed from this connected topology. Subsequently, the locations of CSs are updated sequentially to reduce the average flight distance of the affected customers while maintaining the MST topology to avoid disconnection with any customer.

#### 3.2.3. Delivery Destination Clustering (DDC)

In DDC [17], the authors utilized the k-means clustering method to optimally cluster the delivery destinations and allocate one CS at each centroid of the cluster. The delivery coordinates are generated based on the frequently visited customers’ locations. This process also helps to find the optimal number and locations of CSs, which can extend the drone flight time to cover multiple customers in a single mission. Nevertheless, due to the constrained flight time a large cluster may not be covered by a single CS located at centroid of the cluster. Considering this real-life scenario, DDC also considers necessity of placing additional CSs within a large cluster to successfully execute the mission.

#### 3.2.4. Cloud-Based Drone Navigation and Charging (CDNC)

If a drone chooses the CSs individually without considering any central coordination and presence of other drones, they may encounter congestion to acquire access to the CSs for their battery replenishment. To minimize the congestion at CSs, in [51], the authors proposed the CDNC, which collects the drones’ traffic information and delivers the optimal drone routes using central cloud management systems. The CDNC has three different components: traffic control center, CSs, and drones. Each drone transmits their source location, destination, and average speed to the cloud-assisted traffic control center. According to the collected information, the traffic control center computes the arrival, charging, and waiting time of each drone at a given CS, utilizes the estimated travel time to assign the most suitable path to each drone, and reserves the CS usage time for each drone to minimize the average travel time. The CDNC utilizes Dijkstra’s shortest path algorithm to generate the optimal route for drone, while considering the charging time and waiting time for charging at CSs, and drone flight time required for the end-to-end path. In addition, the CDNC can predict the congestion of drones at CSs utilizing the historical information of drone trajectories. Based on this prediction, the central controller changes the navigation path of the drones in advance to avoid congestion at specific CSs.

Apart from the above charging strategy, recently, researchers are also utilizing the auction-based game theory [45], and deep reinforcement learning [52] to generate a cost-optimized charging scheduling. In these charging strategies, each drone participates in the auction to buy charging timeslots from the CSs. According to the bidding price, the CSs allocate timeslot to the drone to acquire energy replenishment. To improve the transaction security, the communication between CSs and drone is secured by using different energy-efficient consensus mechanism of blockchain technology such as the Internet of things application (IOTA) [46]. IOTA is a consensus protocol in blockchain specially designed for low power Internet of things (IoT) networks and more detailed study can be found in [53].

### 3.3. Security Algorithms Used in Drone Routing

Drone security is currently one of the major research issues in the industry and academia. Due to the widespread use of drones, problems related to security, privacy, regulation, and ownership are simultaneously rising. Numerous applications are highly critical in terms of security, failing to provide which may considerably harm society [54]. Due to the increasing development of e-commerce, high manpower is required to cover the last-mile delivery of products. In such cases, drones can have a huge potential for delivering the product in shorter time. Although drones have huge potential in e-commerce and logistics, when hacked, they can pose a huge threat to the people as well as to the society. For example, hackers might utilize radars to detect and capture sensitive information such as user and product information. The hackers may even modify the delivery address and send the package to another location or to their location. Therefore, certain security guidelines need to be ensured to guarantee secure, effective, and reliable package delivery using drones. To handle major security issues, blockchain-enabled techniques have been widely investigated in the present literature. Blockchain enables storing the previous blocks as hashes in an immutable ledger. All the stakeholders in the system have access to the ledger as a chain. In order to guarantee the consensus between the stakeholders, a new block is added to the existing chain and is copied to all the other nodes [55]. Thus, this structure cannot be manipulated by anonymous attackers. In this section we discuss several techniques to ensure security issues in drone-based delivery systems.

#### 3.3.1. Blockchain-Based Secure Data Transaction Scheme (Covadel)

For designing a drone-based delivery systems, multiple constraints must be considered, such as the physical resource status, energy, and the operational capability of the hardware. For designing drone routing techniques, the algorithm needs to be adaptive in emergency situations to consider different routes due to unexpected events. Thus, if the drone has insufficient energy for the diverted route, it cannot sustain the flight. Considering these issues, in [56], the authors suggested a blockchain-based delivery service for delivering essential materials or medicines in COVID-19 scenario. To enable secure data transaction between drones and between drones and base station, the drones are first categorized in terms of the current resource and computational capacity before being selected for the delivery mission. This categorization phase allows selecting the drones that have sufficient capability in terms of weight, fitness, payload, and battery level. Second, because the network might face congestion while communicating with an increasing number of drones in a particular area, the authors leveraged the fire-fly optimization technique to maximize the quality of service by designing an effective communication approach via time-slot mechanism. Moreover, the authors leveraged the light intensity parameter for optimizing drone communication, which depends on accurate beam positioning. The simulation study shows that the proposed method can maximize service quality by maximizing throughput and minimizing end-to-end delays. 

#### 3.3.2. Blockchain-Powered Privacy-Aware Flight Compliance (PA-NOP)

Of all the major issues in drone-based delivery service system, one of the major concerns is how to control the flights of the drones when all the drones are developed by third-party organizations. In addition, while designing drone routes, it is important to consider that the route excludes unauthorized areas to ensure privacy and security. Moreover, ensuring that the drones for the delivery purpose comply with these rules and regulations is also an important issue to consider. Therefore, in [57], the authors designed a route planning mechanism where they jointly considered the security of the citizens and contract-based policy for collision avoidance to design a secure and reliable drone-based delivery systems. A three-layered architecture is proposed. The first layer defines the flight policy and the predefined route, which are then programmed into the drone. The second layer manages the drone services following the set of guidelines set by the first layer by collecting all the related data regarding the drones and the flight path. The final layer is the blockchain layer which receives all the data from the second layer and enforces the policies required to ensure the security guidelines. Drones that do not follow the preset guidelines by the controllers receive a penalty from the blockchain controller. Thus, security and collision are ensured by following the proposed path planning mechanism. However, this study only considers enforcement of flight policies and excludes communication security.

#### 3.3.3. Blockchain-Based Drone-Enabled Delivery Scheme for Healthcare (GaRuDa)

Secure drone communication is a crucial for ensuring reliability in drone-based delivery systems. For effective route planning from source to destination, drones need to communicate with each other within a particular coverage range. Since the parcel delivery might be in adverse conditions, the communication network may face unexpected situations such as bandwidth limitation, which can cause delay and inaccuracy in the information being exchanged. Incorrect information may cause drones to divert from the planned trajectories and enter unauthorized areas. To address the aforementioned problems, the authors in [58] proposed a secure scheme named GaRuDa, which incorporates the internet of drones and blockchain technology between different stakeholders to enable delivery scheme with a short delay and high efficiency. The proposed framework works in three phases: The first phase, also called as data dissemination phase, consists of the healthcare institutions, staffs, and pharmacies that receive requests for health products from the patients. A hashed key value is used for accessing every record to make the information secure and reliable. In the second phase, the package is delivered by the drone using a 5G-enabled internet channel if they comply with the smart contract conditions. The last phase consists of the consumers, who are the main requesters of the drugs. In particular, the proposed framework uses blockchain-based smart contracts to guarantee trusted operation. The authors consider the inclusion of payment gateway to evaluate the effectiveness of the proposed model with respect to data storage, communication, and computation. The proposed technique can reduce the communication cost when the number of users increases. However, communication interference (co-channel and cross-channel) was not considered in this study.

#### 3.3.4. IDS and Blockchain-Based Delivery Framework for Drone-Delivered Services (DeliveryCoin)

Recently intrusion detection techniques have been emphasized for detecting any external hacker and attackers attempt to penetrate the system [59]. To deliver privacy preserving drone-based delivery systems, the authors in [60] proposed a blockchain-enabled intrusion detection technique to enable package delivery among the drones. The framework consists of five entities, namely, package buyer, package vendor, delivery service, autonomous vehicle, and macro eNB. First, as package buyers, both customer and store center can buy certain packages from package vendor. Next, the package delivery service handles the transactions between the buyer and vendor through blockchain-enabled digital ledger, which is propagated through all the nodes. During the transactions, to detect any false transactions and attacks, machine learning techniques are utilized to ensure that the transactions remain secure inside the framework. The proposed study considers both drones and vehicles as part of the framework. Finally, a terrestrial cellular network is considered to serve the aerial as well as ground users. To achieve a consensus in the blockchain-framework, they employed a drone-based forwarding mechanism named pBFTF, which mainly updates the transactions. Next, in the intrusion detection phase, several machine learning classifiers such as Random Forest, Linear Regression, and Naïve Bayes are utilized to detect network attacks and false transactions. The authors consider the combination of signatures with hash functions to achieve consensus in the delivery framework. The proposed system can detect network attacks and treat them as false transactions. Thus, the overall framework is secured. Moreover, sufficient consensus latency and accuracy are ensured.

## 4. Comparison

In this section, the reviewed algorithms for drone routing in drone-based delivery systems are qualitatively compared with each other for readers to acquire useful information in the three major aspects of trajectory planning, charging, and security algorithms used in drone routing.

### 4.1. Comparison of Trajectory Planning Algorithms

Following the studied scheme in Section 3.1, this subsection demonstrates a relative comparison among the different trajectory strategies taken by the authors in terms of main idea with their respective advantages, limitations, evaluated metrics, performance objective and innovative ideas. Table 2 lists the drone-based delivery techniques that focus on the trajectory design with their respective advantages and limitations. Based on each trajectory scheme discussed earlier for the drone-based delivery systems, most studies focus on either maximizing the flight time to ensure maximum coverage in a single charge or minimizing the traveling distance for delivery. Many of the algorithmic approaches resort to heuristic approaches in the problem formulation. However, machine learning-based approaches are promising performance despite being computationally expensive, which allows for their practical implementation. Despite proving the optimal flight path, none of these schemes considered the impact of weather and various wind conditions on the trajectory formulation.

Table 3 compares the different studied schemes for trajectory technique of drone-based delivery systems in terms of the evaluation metrics, performance objectives, and innovative features. Each of these techniques is evaluated with their respective target parameters, as discussed in Table 3. However, each technique has their uniqueness either in terms of algorithmic solution approaches or formulating the desired objective in a meaningful way. However, a study on complete autonomy in drone-based and multi-drone-based delivery systems, where the drones are working cooperatively, is yet to be conducted.

### 4.2. Comparison of Charging Algorithms

According to the discussion in Section 3.2, in this subsection different charging strategies in drone-based delivery systems are compared with each other. Table 4 lists the objectives, advantages, limitations, innovative features, and constraints considered in the optimization process and the performance metrics of different charging strategies. As provided, the main objective of charging strategies is to maximize the delivery coverage area, drone flight time, and minimize the number of required CSs according to the distribution of delivery destinations by adjusting the positions of CSs to reduce the deployment cost.

According to Table 5, most methods ignored the environmental effects such as the presence of wind, mountain, and external obstacles, to calculate the realistic drone flight time, which is crucial to obtain the optimal locations of CSs. Additionally, an accurate energy consumption model considering drone type, aerodynamics, payload size, and trajectory is required to estimate the optimal flight time. Next, most algorithms, except CBPTCS [11], ignore the restriction for placing the CSs near restricted areas such as rivers, mountains, etc. In the optimization process, all the considered algorithms, except CDNC [51], consider that the CSs have sufficient resources, such as availability of charging pads, and spare fully-charged batteries. Moreover, they ignored the failure possibility of CSs. This assumption may be inaccurate in real-life scenarios, as CSs have limited resources to allocate to drones. Any additional request beyond its capacity may result in drone failure and severe congestion at providing service, which may lead to longer waiting times in CSs. This further increases the flight time of drones and reduces the average delivery time of goods.

### 4.3. Comparison of Security Algorithms in Drone Routing

Based on the discussion in Section 3.3, in this subsection, different security strategies in drone-based delivery systems are compared with each other in terms of their main idea, advantages, limitations, evaluated metrics, performance objectives, and innovative ideas. In Table 6, drone-based delivery techniques that emphasize security issues for drone-based delivery are compared in terms of their main goal, advantages, and limitations. It can be observed that blockchain technology is the most widely used approach to guarantee security and privacy in drone-based delivery systems. The drones being used for delivering packages are generally considered as the nodes in the blockchain. The two major advantages of blockchain technology are immutability and decentralized architecture, ensuring the overall framework is secure. Different approaches have considered a variety of security issues such as intrusion detection, trajectory planning for avoiding restricted areas, and security of user and product data.

Table 7 summarizes the existing techniques in terms of their performance-based features, such as evaluated metrics and innovative ideas. For ensuring security issues via blockchain technology, the security performance of the proposed model was evaluated using several crucial metrics have been considered, such as mining and transaction times, storage cost, and latency of blockchain consensus. We observed that in addition to these parameters, other metrics such as communication and computation overhead, end-to-end delay, and network throughput have also been considered to minimize the overall energy cost to ensure reliable and effective delivery performance. This is mainly because adding an extra payload on the drone causes the battery to drain much faster, which directly affects the overall flight time. Furthermore, energy consumption varies continuously with the dynamic payload weight.

Weather uncertainty and customer demand are some of the major issues that remain underexplored in the existing study. For example, heavy wind and rain can harm the aircraft and would risk the lives of the citizens. This would incur immense penalties on the service providers. Furthermore, in urban areas, the amount of package delivery is extremely high and drones from other service providers as well as the high-rise buildings make it challenging to design routes with minimum flight time. Thus, uncertainty-aware route planning is still an open issue in the field of drone-based logistics. To design an effective and reliable drone-based delivery system, various design aspects need to be considered, which was the goal of this study. Thus, although the existing studies present promising development in this area, further research is needed to address the security concerns in large-scale delivery scenarios and in uncertain conditions.

## 5. Research and Development Challenges

Although there have numerous efforts been made for designing drone routing techniques in drone-based logistics systems, there still exist some crucial challenges introduced by the increasing demand from the customers and uncertain environment conditions that remain to be considered. In this section, we provide some open research challenges to motivate further research in this ever-increasing domain.

### 5.1. Joint Routing and Charging Strategy

Drone route planning is commonly studied problems in drone-based delivery. Although existing literature addresses many issues in drone routing to minimize the average delivery time and maximize the delivery coverage, this area still requires further study. Here, owing to the limited payload capacity, limited battery, environmental effect (i.e., wind, surrounding dynamic, and static obstacles), and restricted flying zone, drone routing problem becomes extremely challenging. With the increased number of depots, number of trucks, number of customers or delivery points, and coverage area, long-range multi-drone routing requires a suitable charging strategy to extend the drone flight time, and enable it to visit multiple nearby delivery points within a single mission [61]. Each drone requires a suitable energy consumption model according to its type, and propulsion power consumption to accurately estimate its flight time based on its traveled trajectory. Thus, the joint consideration of drone routing and charging strategy requires further research by adopting a realistic energy consumption model. Additionally, the placement of optimal number of CSs according to the optimal clustering of delivery locations considering several practical constraints such as restriction in drone payload, placement of CSs, deployment cost, optimizing the number of CSs, and their resources (spare batteries, or charging pad), and congestion at CSs, needs to be considered to address the existing research gap [12].

### 5.2. Dynamic Obstacles

With an increasing number of drones being developed by different service providers, collision avoidance techniques need to be designed more carefully to ensure safe flights and successful parcel delivery and take-off. Thus, drone routes from other service providers should also be considered [14,62]. In [62], the authors proposed a centralized algorithm to find the collision free-path considering a congested flight space owing to the presence of other drones. They also consider a realistic energy consumption model for drones using a data-driven method to estimate the drone battery state of charge. 

In addition, in urban areas, the presence of many high-rise buildings and trees makes the route planning challenging. The consideration of three-dimensional map of urban area incorporating digital twin technology can help create a high-fidelity environment of the real world, which can be utilized to train drones to find the optimal path using different machine learning algorithms. Simultaneously, drone routing considering dynamic obstacles and additional uncertainties can make the algorithm more robust and efficient. Machine learning techniques, such as supervised learning methods, need sequential trajectory information or labeled data to find the optimal trajectory. In such cases, reinforcement learning-based methods can successfully handle dynamic environments through trial-and-error methods without any pre-provided dataset [63].

### 5.3. Topology Control to Reduce Flight Time

Topology control is a major issue for reducing drone flight time. In multi-UAV systems, selecting the path with the most effective route is a challenging issue that must be solved to minimize the overall energy consumption and travel distance [33]. The joint consideration of the topology and routing to select the optimal path is shown to be successful in minimizing delay as well as energy consumption [64,65]. Thus, to meet the excessive parcel delivery requirements, multi-UAV deployment can be a potential solution [66].

### 5.4. Collaborative Truck-Drone Delivery

Collaboration between truck and drones is shown to be successful to address the flight range and payload limitation problem of drones [67,68,69]. In [15], the authors proposed a contactless collaborative truck-drone delivery to prevent the further spread of COVID-19 disease. They formulated a mixed-integer linear programming to minimize the delivery time while considering the energy consumption model of drones. In [70], the authors considered truck-drone collaborative delivery, where truck operation is restricted by street network and drone operation is restricted by its payload capacity and operation range to improve the efficiency of last-mile delivery. Utilizing the trucks helps to plan effective routes for drones to deliver the parcels in a certain area where the customer locations are widespread [69,71] However, multiple problems remain unaddressed, such as partitioning customers between trucks and drones and determining the location of the truck for launching and synchronizing the route planning to ensure reliable drone-based delivery [72]. Thus, routing and scheduling of the drones should be jointly considered to solve these issues [10]. 

However, truck-drone-based delivery has its demerits. The cost of truck is high because it requires a driver and there exists fuel and maintenance cost. To solve these problems, alternatives such as CS installation [73,74,75] and utilization of public transportation network [18,76,77] have been investigated recently, which are a potential research topic. Additionally, the hybrid trunk-drone delivery problem considering the simultaneous deployment of trucks, truck-carried drones, and independent drones to design more robust truck-drone delivery still requires further study to overcome the limitations of limited battery and payload capacity of drones [10,72,78,79].

### 5.5. Uncertainty-Aware Delivery Service

In drone-based delivery service, several uncertain issues exist such as weather conditions, varying payload, drone airspeed, and customer demand [34,48,80]. Thus, not considering such subtle issues results in increased costs and flight time, which deteriorates the overall delivery performance. Such uncertain conditions could result in drone breakdown, which could affect the next parcel deliveries. In such cases, penalties are significantly higher, which increases the overall cost [81]. Additionally, the presence of no-fly zone, the maximum allowable altitude to fly, nighttime, and maximum transportable load are critical safety issues that must be considered. Most existing studies ignore these uncertain conditions during problem formulation, which remains an open issue. Thus, significant consideration should be given to these factors so that in practical scenarios, the developed approach could handle such situations.

### 5.6. Blockchain for Security in Drone-Based Delivery

To ensure successful package delivery, numerous crucial data need to be processed and stored, such as time, delivery location, date of delivery, and user information. This information needs to be stored in a secure system to prevent others from exploiting these data for heinous purposes [82]. Blockchain can be an effective solution to provide a secure and robust framework for storing such information in a secure platform. Thus, more attention should be given to the design of secure delivery systems. Additionally, a secure transaction network is required between drone and commercially deployed CSs to trade energy [46]. To ensure a secure transaction network between drones and CSs, researchers use different blockchain consensus mechanisms. However, such consensus mechanism is required to exchange distributed trusted ledgers with each other in order to validate each transaction. Since drones have a limited battery lifetime, it requires additional energy consumption to share such consensus messages. As a result, the implementation of energy-efficient secure blockchain mechanism requires further study. In particular, the IOTA consensus mechanism can help implement energy-efficient and secure energy trading between drone and CSs [46,83].

### 5.7. Artificial Intelligence-Based Techniques

Recently, artificial intelligence (AI) based techniques have shown significant research attention due to their smart data-driven decision-making capability. To handle the uncertain and dynamic environment conditions, utilizing advanced AI techniques can provide the optimal solution and better performance compared to traditional optimization techniques for the NP-hard problems [6,84,85].

## 6. Conclusions

In this paper, we surveyed existing drone routing algorithms for drone-based delivery systems. First, we summarized and discussed the design issues of drone-based delivery systems. Next, we extensively reviewed the existing algorithms of drone routing in drone-based delivery with a novel taxonomy in terms of three major aspects, namely, trajectory planning, charging, and security. Subsequently, the reviewed algorithms were qualitatively compared in terms of their main idea, advantages, limitations, and detailed performance details. We also summarized and discussed important research challenges to motivate further study in this field.

Drone-based delivery has captured significant attention from both academia and industry. To ensure seamless connectivity and effective parcel delivery performance, several challenges need to be addressed. Although the existing development in this domain presents significant research contributions, with increasing demand and uncertain environmental conditions, the design of drone-based delivery systems grow increasingly complex. Thus, it is crucial to design efficient and reliable drone routing algorithms for drone-based delivery systems by considering practical conditions and uncertainty in the environment. To reach the next step in future parcel delivery, drone-based logistics systems are going to be a crucial building block for enhanced service in the future.

## Figures and Tables

**Table 2:** Comparison of trajectory planning algorithms in drone routing in terms of main idea, advantages, and limitations.

Ref.	Main Idea	Advantages	Limitations
A-Ptr-Net [6]	Drone-based automated trajectory design for optimal delivery path.	Adaptability to new trajectory data regardless of explicit distance matrix. Takes non-linear energy consumption, customer demand and service timeframe into account while calculating optimal route.	Requires large data volume for accurate drone trajectory prediction.
JRCS [12]	For long-haul drone-based delivery mission, simultaneous route and CS location is devised to maximize the safe flight coverage.	This scheme provides maximum delivery coverage (distance) while utilizing the well-placed CSs across the area in a single mission.	Proposed scheme ignores the effect of weather as well as any obstacle that the drone might face along its trajectory.
CRP-TD [15]	A collaboration of truck-drone combination to solve the preassigned delivery problem	The algorithm integrates the K-means clustering and the nearest neighbor strategy to generate an initial solution which helps to reach optimal solutions quickly and accurately.	Stochastic demand for the delivery schedule is not taken into design consideration.
EHTDDP [16]	Formulates a combined truck-drone delivery problem which is addressed by MIP and heuristic approach	The hybrid heuristic based on general variable neighborhood search metaheuristics is able to obtain high-quality solutions for large-size instances with an efficiency rate over 80%.	Only single drone-based delivery scenario is considered. Moreover, the proposed heuristic approach does not guarantee optimal solution
FSTSP-STT [41]	Flight path of the drone is formulated as MDP and minimizing the flight time is prioritized.	This model incorporates stochastic traffic conditions and works with artificially generated data to demonstrate the algorithmic working efficiency. Moreover, real-time data were also used for further validation.	Proposed approach demonstrates higher computational time and flight data than other benchmarks.
RL-TDCD [42]	A combined delivery method of truck and drone is proposed where clustering of the designated delivery point and routing plan is designed.	This algorithm has shorter execution time compared to the other heuristic approaches and reduces the overall drone energy consumption.	The problem formulation ignores the time required for charging or swapping batteries of drones as well as the loading/unloading parcel time.

**Table 3:** Comparison of trajectory planning algorithms in drone routing in terms of evaluated metrices, performance objective, and innovative features.

Ref.	Evaluated Metrics	Performance Objective	Innovative Features
A-Ptr-Net [6]	Time of customer nodes, trajectory optimality gap, effect of attention mechanism, energy gap.	Minimizing route distance by finding optimal route path.	Attention mechanism on the decoder end to adjust the trajectory information.
JRCS [12]	Average delivery time, algorithm computation time, delivery area coverage ratio, drone travel distance, the number of customers served, and energy consumption.	Maximizing drone flight distance with respect to the drone CS.	Maximum flight distance and safe flight distance for the drone-based delivery system is considered for problem formulation.
CRP-TD [15]	Instances, number of drones, drone speed.	Minimizing the delivery time.	K-means clustering and nearest neighbor strategy to generate an initial solution.
EHTDDP [16]	Instances, service time, number of distribution centers.	Minimized delivery time and cost.	MIP formulation yields better linear relaxation bounds than other benchmarks.
FSTSP-STT [41]	Variation of travel time, average delivery time.	Minimizing the stochastic travel time of the drone for delivery.	DQN and A2C are combined and used to address the problem of dimensionality.
RL-TDCD [42]	Average travel distance, travel time, average running time, number of clusters, convergence rate.	Minimizing the average travel distance	Provide last mile parcel delivery using a combined truck and drone system.

**Table 4:** Comparison of charging algorithms in drone routing in terms of main idea, advantages, and limitations.

Ref.	Main Idea	Advantages	Limitations
CBPTCS [11]	Minimize the average delivery time to customers.	The restriction of placing CSs location based on the restricted area is considered in the optimization process along with the precise calculation of drone’s flight time.	The limited battery resources of CSs are not considered in the optimization process. Additionally, CBPTCS relocates a CS based on the numerical computation, whereas an analytical solution is required for faster computation.
DDC [17]	Maximizing the delivery area through optimal clustering of delivery locations and minimizing the number of required CSs.	Through optimal *k*-means clustering of delivery locations, it jointly optimizes the CSs locations to extend the drones flight time and coverage area.	The locations of restricted areas to place CSs is ignored. Additionally, the coverage area can be improved by minimizing the gap and overlap between two neighboring cluster centroids.
ODCS [44]	Obtain the optimal number of CSs and their location.	The recursive removal of CSs according to the imposed constraint of CS coverage ratio, achieves the optimal location and quickly minimize the number of CSs to cover a large area.	This method only considers the CSs location adjustment according to the fixed threshold flight time of drones. However, drone flight times may vary according to their trajectories, and environmental influences such as wind. Thus, drone energy consumption model is required to obtain realistic results in the simulation environment.
CDNC [51]	Minimize the congestion at CSs to provide smooth charging service to drones and minimize the drones waiting time at CSs using Dijkstra’s shortest path algorithm.	This approach utilizes the global knowledge to generate more stable optimal path for drones, which reduces congestion at CSs and waiting time for charging.	The optimal deployment of CSs according to drone trajectory is not considered. In trajectory planning, the physical collision of drones and environmental factors were ignored

**Table 5:** Comparison of charging algorithms in drone routing in terms of innovative features, performance metrics, and optimization constraints.

Ref.	Innovative Features	Performance Metrics	Optimization Constraints
CBPTCS [11]	Jointly considers collaboration between public transport, drones, and CSs to maximize drones flight time to server rural areas customer.	Average delivery time, number of required CSs, and average drone flight time	Limited number of CSs, threshold distance between CSs according to drone flight time, and connectivity between CSs and all customers.
DDC [17]	Utilizes *k*-means algorithm to cluster the frequently delivery locations and placing the CSs.	Coverage ratio, and number of CSs	Limited number of CSs.
ODCS [46]	Utilizes the MST to find the optimal flight path and placement of CSs to extend the drone flight time.	Delivery coverage ratio, and number of required CSs.	Limited parcel weight, distance between two CSs according to the flight time of a drone, and threshold coverage ratio of CSs.
CDNC [51]	A cloud-based multiple drones navigation and charging utilizing global information about drone’s trajectory, flight time, and status of CSs.	Number of CSs, average drone flight time, and average utilization of CSs	Central cloud server must have connectivity with all drones and CSs to obtain the global knowledge.

**Table 6:** Comparison of security algorithms in drone routing in terms of main idea, advantages, and limitations.

Ref.	Main Idea	Advantages	Limitations
Covadel [56]	The scheme considers queuing schedule for the delivery of the goods with minimal communication overhead.	Effect of weather on drone energy consumption and charging mechanism were ignored.	A secure collision avoidance mechanism for drone-based delivery is designed based on light intensity.
PA-NOP [57]	Study of collision avoidance among multiple drones from different organizations to provide secure delivery service.	The proposed scheme provides a secure delivery flight operation by introducing blockchain mechanism.	Scalability of the proposed mechanism is not guaranteed.
GaRuDa [58]	Considered the security aspects for healthcare-based product delivery using Internet of drones.	The system can offer better scalability and minimize the storage cost for blockchain data storage.	The co-channel and cross-channel interference issues are overlooked.
Delivery-Coin [60]	Proposed an intrusion detection technique to provide privacy preserving drone-based delivery systems.	Proposed framework has the lower consensus latency with respect to other conventional schemes.	Security issues of edge computing in the proposed framework were ignored.

**Table 7:** Comparison of security algorithms in drone routing in terms of evaluated metrics, performance objective, and innovative features.

Ref.	Evaluated Metrics	Performance Objective	Innovative Features
Covadel [56]	Transaction time, mining time, communication cost, computation cost, average network throughput, and end-to-end delay.	Provides secure and quality of service (QoS) to users of drone-based delivery	Decoupled blockchain-based secure delivery mechanism.
PA-NOP [57]	Execution time, validation time, and service delivery time.	Maximizing the scalability of the system.	Jointly considered security and collision avoidance.
GaRuDa [58]	Storage cost, computation cost, and communication cost.	Minimize the blockchain storage cost.	Latency issue has been considered by considering the 5G-enabled tactile internet.
Delivery-Coin [60]	Latency of blockchain consensus, communication overhead, attack classification accuracy.	Minimizing the overall latency of blockchain consensus and accuracy.	Combined intrusion system and blockchain technology to secure customer and product data.

## Data Availability

Not applicable.

## References

[1] Pitney Bowes Parcel Shipping Index Reports Continued Growth as Global Parcel. https://www.pitneybowes.com/au/newsroom/press-releases/pitney-bowes-parcel-shipping-index-reports-continued-growth-as-global-parcel.html.

[2] Jeon A., Kang J., Choi B., Kim N., Eun J., Cheong T. (2021). Unmanned Aerial Vehicle Last-Mile Delivery Considering Backhauls. IEEE Access.

[3] Zhu H., Dou S., Qiu Y. (2019). Joint model for last-mile delivery service selection in china: Evidence from a cross-nested logit study. IEEE Access.

[4] Benarbia T., Kyamakya K. (2022). A literature review of drone-based package delivery logistics systems and their implementation feasibility. Sustainability.

[5] Nguyen D.D., Rohacs J., Rohacs D. (2021). Autonomous flight trajectory control system for drones in smart city traffic management. ISPRS Int. J. Geo-Inf..

[6] Kong F., Li J., Jiang B., Wang H., Song H. (2022). Trajectory Optimization for Drone Logistics Delivery via Attention-Based Pointer Network. IEEE Trans. Intell. Transp. Syst..

[7] Jeong H.Y., Song B.D., Lee S. (2021). The Flying Warehouse Delivery System: A Quantitative Approach for the Optimal Operation Policy of Airborne Fulfillment Center. IEEE Trans. Intell. Transp. Syst..

[8] Gao W., Luo J., Zhang W., Yuan W., Liao Z. (2020). Commanding Cooperative UGV-UAV with Nested Vehicle Routing for Emergency Resource Delivery. IEEE Access.

[9] Savuran H., Karakaya M. (2016). Efficient route planning for an unmanned air vehicle deployed on a moving carrier. Soft Comput..

[10] Wang D., Hu P., Du J., Zhou P., Deng T., Hu M. (2019). Routing and Scheduling for Hybrid Truck-Drone Collaborative Parcel Delivery With Independent and Truck-Carried Drones. IEEE Internet Things J..

[11] Huang H., Savkin A.V. (2021). Deployment of Charging Stations for Drone Delivery Assisted by Public Transportation Vehicles. IEEE Trans. Intell. Transp. Syst..

[12] Arafat M.Y., Moh S. (2022). JRCS: Joint Routing and Charging Strategy for Logistics Drones. IEEE Internet Things J..

[13] Dorling K., Heinrichs J., Messier G.G., Magierowski S. (2017). Vehicle Routing Problems for Drone Delivery. IEEE Trans. Syst. Man, Cybern. Syst..

[14] Shen K., Shivgan R., Medina J., Dong Z., Rojas-Cessa R. (2022). Multidepot Drone Path Planning With Collision Avoidance. IEEE Internet Things J..

[15] Wu G., Mao N., Luo Q., Xu B., Shi J., Suganthan P.N. (2022). Collaborative Truck-Drone Routing for Contactless Parcel Delivery During the Epidemic. IEEE Trans. Intell. Transp. Syst..

[16] Freitas J.C., Penna P.H.V., Toffolo T.A.M. (2022). Exact and heuristic approaches to Truck-Drone Delivery Problems. EURO J. Transp. Logist..

[17] Ahmadon M.A.B., Yamaguchi S. Cluster-Based Positioning Method of Drone Charging Station for Enlargement of Delivery Area. Proceedings of the 2021 IEEE International Conference on Consumer Electronics (ICCE).

[18] Trotta A., Andreagiovanni F.D., Di Felice M., Natalizio E., Chowdhury K.R. When UAVs Ride a Bus: Towards Energy-efficient City-scale Video Surveillance. Proceedings of the IEEE INFOCOM 2018—IEEE Conference on Computer Communications.

[19] Seo S.H., Won J., Bertino E., Kang Y., Choi D. A security framework for a drone delivery service. Proceedings of the 2nd Workshop on Micro Aerial Vehicle Networks, Systems, and Applications for Civilian Use.

[20] Singh M., Aujla G.S., Bali R.S., Vashisht S., Singh A., Jindal A. Blockchain-enabled secure communication for drone de-livery: A case study in COVID-like scenarios. Proceedings of the 2nd ACM MobiCom Workshop on Drone Assisted Wireless Communications for 5G and beyond.

[21] Rajabi M.S., Beigi P., Aghakhani S. (2022). Drone Delivery Systems and Energy Management: A Review and Future Trends. arXiv.

[22] Moshref-Javadi M., Winkenbach M. (2021). Applications and Research avenues for drone-based models in logistics: A classification and review. Expert Syst. Appl..

[23] Yoo W., Yu E., Jung J. (2018). Drone delivery: Factors affecting the public’s attitude and intention to adopt. Telemat. Informatics.

[24] Madani B., Ndiaye M. (2022). Hybrid Truck-Drone Delivery Systems: A systematic literature review. IEEE Access.

[25] Pasha J., Elmi Z., Purkayastha S., Fathollahi-Fard A.M., Ge Y.E., Lau Y.Y., Dulebenets M.A. (2022). The Drone Scheduling Problem: A Systematic State-of-the-Art Review. IEEE Trans. Intell. Transp. Syst..

[26] Khalil A., Shankar A., Bodhi R., Behl A., Ferraris A. (2022). Why Do People Resist Drone Food Delivery Services? An Innovation Resistance Theory Perspective. IEEE Trans. Eng. Manag..

[27] Raivi A.M., Moh S. Trajectory Planning Techniques in Urban Air Mobility: A Comparative Survey. Proceedings of the 11th International Conference on Smart Media and Applications (SMA 2022).

[28] Singh G., Sharma S., Tandon A., Kaur P. (2022). Drone Food Delivery: A Solution to Crowding During the Global COVID-19 Pandemic. IEEE Trans. Eng. Manag..

[29] Macrina G., Di Puglia Pugliese L., Guerriero F., Laporte G. (2020). Drone-aided routing: A literature review. Transp. Res. Part C Emerg. Technol..

[30] Kornatowski P.M., Bhaskaran A., Heitz G.M., Mintchev S., Floreano D. (2018). Last-Centimeter Personal Drone Delivery: Field Deployment and User Interaction. IEEE Robot. Autom. Lett..

[31] Huang C., Ming Z., Huang H. (2022). Drone Stations-Aided Beyond-Battery-Lifetime Flight Planning for Parcel Delivery. IEEE Trans. Autom. Sci. Eng..

[32] Huda S.M.A., Moh S. (2022). Survey on computation offloading in UAV-Enabled mobile edge computing. J. Netw. Comput. Appl..

[33] Alam M.M., Arafat M.Y., Moh S., Shen J. (2022). Topology control algorithms in multi-unmanned aerial vehicle networks: An extensive survey. J. Netw. Comput. Appl..

[34] Sorbelli F.B., Coro F., Das S.K., Pinotti C.M. (2021). Energy-Constrained Delivery of Goods with Drones under Varying Wind Conditions. IEEE Trans. Intell. Transp. Syst..

[35] Peng K., Du J., Lu F., Sun Q., Dong Y., Zhou P., Hu M. (2019). A Hybrid Genetic Algorithm on Routing and Scheduling for Vehicle-Assisted Multi-Drone Parcel Delivery. IEEE Access.

[36] Alkouz B., Abusafia A., Lakhdari A., Bouguettaya A. (2022). In-Flight Energy-Driven Composition of Drone Swarm Services. IEEE Trans. Serv. Comput..

[37] Chen K.W., Xie M.R., Chen Y.M., Chu T.T., Lin Y.B. (2022). DroneTalk: An Internet-of-Things-Based Drone System for Last-Mile Drone Delivery. IEEE Trans. Intell. Transp. Syst..

[38] Pei Z., Fang T., Weng K., Yi W. (2022). Urban On-Demand Delivery via Autonomous Aerial Mobility: Formulation and Exact Algorithm. IEEE Trans. Autom. Sci. Eng..

[39] Murray C.C., Chu A.G. (2015). The flying sidekick traveling salesman problem: Optimization of drone-assisted parcel delivery. Transp. Res. Part C Emerg. Technol..

[40] Wang Z., Sheu J.B. (2019). Vehicle routing problem with drones. Transp. Res. Part B Methodol..

[41] Liu Z., Li X., Khojandi A. (2022). The flying sidekick traveling salesman problem with stochastic travel time: A reinforcement learning approach. Transp. Res. Part E Logist. Transp. Rev..

[42] Wu G., Fan M., Shi J., Feng Y. (2021). Reinforcement Learning based Truck-and-Drone Coordinated Delivery. IEEE Trans. Artif. Intell..

[43] Resat H.G. (2020). Design and Analysis of Novel Hybrid Multi-Objective Optimization Approach for Data-Driven Sustainable Delivery Systems. IEEE Access.

[44] Huang H., Savkin A.V. (2020). A Method of Optimized Deployment of Charging Stations for Drone Delivery. IEEE Trans. Transp. Electrif..

[45] Hassija V., Saxena V., Chamola V. (2020). Scheduling drone charging for multi-drone network based on consensus time-stamp and game theory. Comput. Commun..

[46] Hassija V., Chamola V., Krishna D.N.G., Guizani M. (2020). A Distributed Framework for Energy Trading between UAVs and Charging Stations for Critical Applications. IEEE Trans. Veh. Technol..

[47] Huda S.M.A., Arafat M.Y., Moh S. (2022). Wireless Power Transfer in Wirelessly Powered Sensor Net works: A Review of Recent Progress. Sensors.

[48] Sawadsitang S., Niyato D., Tan P.S., Wang P. (2019). Joint Ground and Aerial Package Delivery Services: A Stochastic Optimization Approach. IEEE Trans. Intell. Transp. Syst..

[49] Zhang J., Campbell J.F., Sweeney D.C., Hupman A.C. (2021). Energy consumption models for delivery drones: A comparison and assessment. Transp. Res. Part D Transp. Environ..

[50] Torabbeigi M., Lim G.J., Kim S.J. (2020). Drone Delivery Scheduling Optimization Considering Payload-induced Battery Consumption Rates. J. Intell. Robot. Syst. Theory Appl..

[51] Kim J., Kim S., Jeong J., Kim H., Park J.S., Kim T. (2019). CBDN: Cloud-Based Drone Navigation for Efficient Battery Charging in Drone Networks. IEEE Trans. Intell. Transp. Syst..

[52] Shin M., Kim J., Levorato M. (2019). Auction-Based Charging Scheduling with Deep Learning Framework for Multi-Drone Networks. IEEE Trans. Veh. Technol..

[53] Alshaikhli M., Elfouly T., Elharrouss O., Mohamed A., Ottakath N. (2022). Evolution of Internet of Things from Blockchain to IOTA: A Survey. IEEE Access.

[54] Hassija V., Chamola V., Agrawal A., Goyal A., Luong N.C., Niyato D., Yu F.R., Guizani M. (2021). Fast, Reliable, and Secure Drone Communication: A Comprehensive Survey. IEEE Commun. Surv. Tutorials.

[55] El Azzaoui A., Singh S.K., Pan Y., Park J.H. (2020). Block5GIntell: Blockchain for AI-Enabled 5G Networks. IEEE Access.

[56] Singh M., Aujla G.S., Bali R.S., Batth R.S., Singh A., Vashisht S., Jindal A. (2022). CovaDel: A blockchain-enabled secure and QoS-aware drone delivery framework for COVID-like pandemics. Computing.

[57] Rahman M.S., Khalil I., Atiquzzaman M. (2021). Blockchain-Powered Policy Enforcement for Ensuring Flight Compliance in Drone-Based Service Systems. IEEE Netw..

[58] Gupta R., Bhattacharya P., Tanwar S., Kumar N., Zeadally S. (2021). GaRuDa: A Blockchain-Based Delivery Scheme Using Drones for Healthcare 5.0 Applications. IEEE Internet Things Mag..

[59] Huda S.M.A., Mahir E.M., Tanvir A.A., Moh S. Evaluation of Machine Learning Models for Detecting Network-Based Intrusions. Proceedings of the 11th International Conference on Smart Media and Applications (SMA 2022).

[60] Ferrag M.A., Maglaras L. (2019). Deliverycoin: An IDS and blockchain-based delivery framework for drone-delivered services. Computers.

[61] Alyassi R., Khonji M., Karapetyan A., Chau S.C.K., Elbassioni K., Tseng C.M. (2022). Autonomous Recharging and Flight Mission Planning for Battery-Operated Autonomous Drones. IEEE Trans. Autom. Sci. Eng..

[62] Lee S., Hong D., Kim J., Baek D., Chang N. (2022). Congestion-aware Multi-Drone Delivery Routing Framework. IEEE Trans. Veh. Technol..

[63] Alam M.M., Moh S. Survey on Neighbor Discovery and Beam Alignment in mmWave-Enabled UAV Swarm Networks. Proceedings of the 11th International Conference on Smart Media and Applications (SMA 2022).

[64] Alam M.M., Moh S. (2022). Survey on Q-Learning-Based Position-Aware Routing Protocols in Flying Ad Hoc Networks. Electronics.

[65] Alam M.M., Moh S. (2022). Joint topology control and routing in a UAV swarm for crowd surveillance. J. Netw. Comput. Appl..

[66] Kuru K., Ansell D., Khan W., Yetgin H. (2019). Analysis and Optimization of Unmanned Aerial Vehicle Swarms in Logistics: An Intelligent Delivery Platform. IEEE Access.

[67] Kim S., Moon I. (2019). Traveling salesman problem with a drone station. IEEE Trans. Syst. Man, Cybern. Syst..

[68] Luo Q., Wu G., Ji B., Wang L., Suganthan P.N. (2022). Hybrid Multi-Objective Optimization Approach With Pareto Local Search for Collaborative Truck-Drone Routing Problems Considering Flexible Time Windows. IEEE Trans. Intell. Transp. Syst..

[69] Liu Y., Liu Z., Shi J., Wu G., Pedrycz W. (2021). Two-Echelon Routing Problem for Parcel Delivery by Cooperated Truck and Drone. IEEE Trans. Syst. Man, Cybern. Syst..

[70] Bai X., Cao M., Yan W., Ge S.S. (2020). Efficient Routing for Precedence-Constrained Package Delivery for Heterogeneous Vehicles. IEEE Trans. Autom. Sci. Eng..

[71] Huang H., Savkin A.V., Huang C. (2020). Round Trip Routing for Energy-Efficient Drone Delivery Based on a Public Transportation Network. IEEE Trans. Transp. Electrif..

[72] Das D.N., Sewani R., Wang J., Tiwari M.K. (2021). Synchronized Truck and Drone Routing in Package Delivery Logistics. IEEE Trans. Intell. Transp. Syst..

[73] Hong I., Kuby M., Murray A.T. (2018). A range-restricted recharging station coverage model for drone delivery service planning. Transp. Res. Part C Emerg. Technol..

[74] Shavarani S.M., Mosallaeipour S., Golabi M., İzbirak G. (2019). A congested capacitated multi-level fuzzy facility location problem: An efficient drone delivery system. Comput. Oper. Res..

[75] Shavarani S.M., Nejad M.G., Rismanchian F., Izbirak G. (2018). Application of hierarchical facility location problem for optimization of a drone delivery system: A case study of Amazon prime air in the city of San Francisco. Int. J. Adv. Manuf. Technol..

[76] Yoo H.D., Chankov S.M. Drone-delivery Using Autonomous Mobility: An Innovative Approach to Future Last-mile De-livery Problems. Proceedings of the 2018 IEEE International Conference on Industrial Engineering and Engineering Management (IEEM).

[77] Huang H., Savkin A.V., Huang C. (2021). Reliable Path Planning for Drone Delivery Using a Stochastic Time-Dependent Public Transportation Network. IEEE Trans. Intell. Transp. Syst..

[78] Gomez-Lagos J., Candia-Vejar A., Encina F. (2021). A New Truck-Drone Routing Problem for Parcel Delivery Services Aided by Parking Lots. IEEE Access.

[79] Huang H., Savkin A.V., Huang C. (2021). Drone Routing in a Time-Dependent Network: Toward Low-Cost and Large-Range Parcel Delivery. IEEE Trans. Ind. Inform..

[80] Hamdi A., Salim F.D., Kim D.Y., Ghari Neiat A., Bouguettaya A. (2021). Drone-as-a-Service Composition Under Uncertainty. IEEE Trans. Serv. Comput..

[81] Sawadsitang S., Niyato D., Tan P.S., Wang P., Nutanong S. (2021). Shipper Cooperation in Stochastic Drone Delivery: A Dynamic Bayesian Game Approach. IEEE Trans. Veh. Technol..

[82] Gumaei A., Al-Rakhami M., Hassan M.M., Pace P., Aloi G., Lin K., Fortino G. (2021). Deep Learning and Blockchain with Edge Computing for 5G-Enabled Drone Identification and Flight Mode Detection. IEEE Netw..

[83] Hassija V., Chamola V., Garg S., Krishna D.N.G., Kaddoum G., Jayakody D.N.K. (2020). A Blockchain-Based Framework for Lightweight Data Sharing and Energy Trading in V2G Network. IEEE Trans. Veh. Technol..

[84] Huda S.M.A., Moh S. Transfer Learning Algorithms in Unmanned Aerial Vehicle Networks: A Comprehensive Review. Proceedings of the 11th International Conference on Smart Media and Applications (SMA 2022).

[85] Chen X., Wu S., Shi C., Huang Y., Yang Y., Ke R., Zhao J. (2020). Sensing Data Supported Traffic Flow Prediction via Denoising Schemes and ANN: A Comparison. IEEE Sens. J..

